# Early Language Development in Infants and Toddlers with Hemato-Oncological Diseases: Preliminary Outcomes of a Shared Reading Intervention

**DOI:** 10.3390/diseases14060193

**Published:** 2026-05-29

**Authors:** Giusy Melcarne, Roberta Maria Incardona, Giulia Marangon, Silvia Sorbara, Alessandra Biffi, Marta Tremolada

**Affiliations:** 1 AIL Padova ODV, 35124 Padova, Italy; robertamaria.incardona@phd.unipd.it; 2 Pediatric Hematology, Oncology and Stem Cell Transplant Center, Department of Woman’s and Child’s Health, University of Padua, 35127 Padova, Italysilvia.sorbara@unipd.it (S.S.); alessandra.biffi@unipd.it (A.B.); marta.tremolada@unipd.it (M.T.); 3 Department of Development and Social Psychology, University of Padua, 35131 Padova, Italy; 4 ANVOLT, Onlus Association, 20158 Milano, Italy

**Keywords:** language, development, communication, cancer, infants, toddlers

## Abstract

Introduction: Children diagnosed with hemato-oncological cancers need intensive medical treatments and prolonged hospitalizations, which are associated with increased risk of impairment across multiple neurodevelopmental domains, particularly when exposure occurs within the first three years of life. Objective: This pilot study aimed to explore early language performance in children under 36 months of age hospitalized in a pediatric hemato-oncology unit and to preliminarily investigate changes over time and potential associations with an early speech–language stimulation intervention based on shared reading. Specifically, the study investigated differences between language comprehension and production, as well as variations in linguistic outcomes according to gender and pathology type (liquid vs. solid). Methods: The study employed a sample of 29 children aged 2 to 36 months (M = 20.76, SD = 9.52). Baseline linguistic assessment was conducted using observational measures across multiple language domains, including lexical development (PinG), morphosyntactic abilities (PCGO, GALS), and articulatory skills (BAMF), together with an evaluation of general cognitive functioning (Griffiths). The intervention consisted of in-person shared reading sessions combined with concurrent parental counseling. Exploratory analyses were performed to examine changes over time and group-related differences. Results: Among the sample, lexical comprehension exceeded production (*p* < 0.001). Sex differences emerged only for lexical comprehension, with males performing worse than females (*p* = 0.048). Comparisons by diagnosis showed that children with solid tumors had significantly better articulation than those with hematologic malignancies (*p* = 0.018), with a trend toward higher morphosyntactic production. Longitudinal analyses on 13 children re-evaluated after six months of weekly shared reading intervention showed potential improvements in articulation, lexical production, and morphosyntactic production (all *p* < 0.01). Conclusions: These preliminary findings suggest that children in pediatric hemato-oncology settings may be vulnerable to expressive language difficulties. Shared reading interventions may represent a promising supportive approach for early language stimulation; however, further studies with larger samples and controlled designs are needed to better understand their potential contribution and effectiveness.

## 1. Introduction

The development of the central nervous system (CNS) is highly sensitive to environmental input, with critical periods of heightened plasticity shaping synaptic connectivity and later cognitive outcomes [1,2]. Adverse conditions such as social deprivation or prolonged hospitalization may interfere with neurodevelopment, with language acquisition appearing particularly vulnerable [3,4]. Children undergoing intensive treatment for hemato-oncological conditions, such as solid and hematological malignancies, face multiple risk factors for communicative and linguistic delays, including treatment-related neurotoxicity, prolonged hospitalization, and reduced social interaction [5,6,7,8]. These effects are particularly evident in children under 36 months, who may show a dissociation between receptive and expressive language abilities [9,10]. From a developmental perspective, these difficulties are consistent with bioecological and experience-dependent models, which emphasize the interaction between biological vulnerability and environmental input in shaping early communication skills [11,12,13,14]. Reduced caregiver interaction and limited linguistic stimulation during hospitalization may further compromise expressive language development [4,5,6,7]. Although language difficulties in this population are well recognized, the literature remains limited and methodologically heterogeneous, with most studies focusing on older children or survivors, while infants, toddlers, and children with hematological malignancies remain underrepresented [15,16,17,18]. Moreover, expressive and receptive language domains are rarely examined in parallel. Despite clinical guidelines recommending systematic involvement of speech–language therapists (SLTs), their role in pediatric hemato-oncology remains largely restricted to feeding and swallowing management, with limited attention to communication and language development [19,20,21]. Evidence suggests that enriched, responsive environments can support neuroplasticity and language development through increased caregiver interaction and linguistic input [11,12,13,14]. Within this framework, shared book reading represents a feasible and developmentally appropriate intervention during hospitalization, yet its application in this context remains underexplored, highlighting a relevant gap and providing the rationale for the present study [22,23].

### 1.1. Shared Book Reading in Contexts of Medical Isolation

Shared reading is a well-established educational and communicative practice known to foster early language development [23]. As an interactive activity between an adult and a child, it promotes exposure to rich vocabulary, complex syntactic constructions, and coherent narrative structures, thereby supporting both receptive and expressive language growth. This dialogic interaction may foster metalinguistic awareness, narrative skills, and cognitive flexibility [24,25,26]. In hospital settings, shared reading may provide structured linguistic input and joint attention opportunities, while also fostering the bond between caregiver and child and offering emotional comfort and a sense of normalcy. Emerging evidence further suggests that integrating meaningful motor gestures during shared reading enhances vocabulary acquisition [23]. When gestures are systematically paired with corresponding words, children show greater vocabulary growth compared to traditional exposure, likely due to embodied learning mechanisms that strengthen semantic encoding and memory consolidation [24,27]. Similarly, during periods of social isolation (e.g., the COVID-19 pandemic), regular shared reading has been associated with better language outcomes, suggesting a possible protective role in restricted environments [5].

### 1.2. Research Aims

This pilot study aimed to explore early language performance in children under 36 months hospitalized in a pediatric hemato-oncology unit. Differences between comprehension and production, as well as variations by sex and diagnosis (liquid vs. solid tumors), were investigated. The study also preliminarily explored longitudinal changes associated with an early shared-reading intervention.

## 2. Materials and Methods

### 2.1. Participants

Participants were recruited from the Pediatric Hemato-Oncology Unit of Padua’s Paediatric Hospital. Inclusion criteria were:

- diagnosis of a pediatric hemato-oncological malignancy;
- age between 0–36 months at the time of diagnosis;
- follow up at the pediatric department of the Hematology–Oncology Clinic of Padua’s Paediatric Hospital;
- being native Italian speakers (L1).

Patients with a diagnosis of neurodevelopmental disorders were excluded. For group comparisons, diagnoses were classified into hematologic malignancies (e.g., leukemias) and solid tumors (e.g., low-grade glioma).

### 2.2. Study Design

The study was designed as a pilot study approved by the Padua Hospital Ethical Committee on 16 September 2024 (code: 6054/AO/24). It was conducted in the pediatric ward of the Hematology–Oncology Clinic within the Department of Children’s and Women’s Health (University of Padua) during the 2024–2025 period as part of the Stai Bene 3.0 Plus! and the Stai Bene 4.0 projects, funded by AIL Padova ODV and the Italian Ministry of Labor and Social Policies. The projects aimed to promote physical, mental, and relational well-being of young patients throughout their entire course of treatment, going beyond medical care by integrating specialized multidisciplinary support, such as speech therapy, physical therapy, nutritional support, and psychological counseling. The children and their parents were invited to participate in the study by the clinic’s clinical psychologist, who had already established a therapeutic relationship with the families through previous counseling and support sessions. After obtaining informed consent, the psychologist introduced the SLT during the families’ hospital stay, whether in the day hospital or inpatient ward, at the time of the cancer diagnosis. All participating caregivers spoke Italian fluently.

Initial assessments were scheduled in consultation with the caregivers. Participants were assessed at the beginning and end of the active treatment phase. The follow-up reassessment was conducted after a minimum of six months of intervention.

### 2.3. Measures

Firstly, a direct assessment was conducted by an expert psychologist using the Griffiths tests and by an expert speech and language therapist through the “Parole in Gioco (PinG)” and “Prova di Comprensione Grammaticale con Oggetti (PCGO)” tests. These tests were designed with age-, gender- and language-appropriate norms, allowing for a meaningful comparison between the patients’ performance and that of typically developing children in the general population. This approach provides a valid benchmark for identifying potential developmental or functional delays. Then, scores were assigned by completing the Brief Assessment of Motor Function (BAMF) scale to assess the child’s articulatory performance and the “Griglie di Analisi del Linguaggio Spontaneo” (GALS) scale to evaluate morphosyntactic production.

#### 2.3.1. Griffiths—Language and Communication Subscale

The Griffiths III scale provides a comprehensive assessment of child development, including intellectual functioning and cognitive growth. Its Language and Communication subscale (B) evaluates overall language skills, covering expressive and receptive abilities as well as syntactic, semantic, and pragmatic components, and includes measures of memory. The Italian adaptation demonstrates excellent psychometric properties, with high internal consistency (α = 0.83–0.99), strong test–retest reliability (0.96–0.99), and robust construct validity. Age- and gender-specific norms allow for the calculation of age-adjusted scores for direct comparison with peers [28].

#### 2.3.2. Parole in Gioco (PinG)

The PinG is a direct observation tool designed to assess language development in children approximately 19–37 months of age. It evaluates both the comprehension and production of nouns and verbs through engaging color photographs, which facilitate word elicitation. Each item correctly produced or understood is scored with one point, and raw scores are then converted into percentiles to allow for standardized comparisons. The PinG demonstrates strong psychometric properties, with excellent internal consistency (Cronbach’s α ≥ 0.80) for most subtests and good test–retest reliability. Repeatability is highest for the production subtests, which also show the strongest correlations with the other subtests, highlighting their sensitivity in assessing early expressive language abilities [29].

#### 2.3.3. Prova di Comprensione Grammaticale con Oggetti (PCGO)

The PCGO is a direct observation tool designed to assess grammatical comprehension in children aged 18–48 months. It evaluates comprehension of simple sentences using familiar three-dimensional objects, while children respond by identifying or acting out the sentences. Each correctly performed item is awarded one point, and raw scores are then converted into percentiles. The tool is highly suitable for young children and those with developmental disorders, demonstrating excellent internal consistency across all score categories (Total Score α = 0.91; Nuclear Phrases α = 0.88; Grammatical Phrases α = 0.90), supporting its reliability in clinical and research settings [30].

#### 2.3.4. Griglie di Analisi del Linguaggio Spontaneo

The GALS is a qualitative tool for assessing children’s spontaneous language production and morphosyntactic development [31]. The examiner evaluates elements such as the use of articles, pronouns, prepositions, verb and noun inflection, syntactic complexity, and discourse coherence. Children are assigned a score from 0 to 5, corresponding to developmental stages ranging from pre-linguistic (0) to advanced morphosyntactic level II (5). As the GALS is a qualitative observational coding system based on developmental levels of spontaneous language production, internal consistency indices are not applicable.

#### 2.3.5. Brief Assessment of Motor Function—Articulation

The BAMF assesses motor and oral motor skills in children across five domains: lower and upper extremity gross motor, upper extremity fine motor, oral motor articulation, and oral motor swallowing. Each task is observed and rated on a scale from 0 to 10, reflecting developmental progression. The oral motor articulation scale ranges from pre-speech vocalizations (0) to fully articulated sentences (10), capturing key milestones related to vocabulary, sentence length, and sound accuracy. The scores provide a quick and reliable overview of the child’s motor and language skills rather than focusing on deficits. The BAMF has demonstrated good psychometric properties in previous validation studies, including high inter-rater (0.94) and intra-rater (0.95) reliability for its motor subscales, as well as content validity established through expert panel review. As the scale has not yet been formally translated and validated in Italian, an Italian version independently derived from the original English instrument reported by Cintas et al. [32] was used, following a direct translation procedure for clinical and research purposes. The translation was reviewed by the research team to verify its conceptual equivalence.

### 2.4. Procedure

The children participated in an early language stimulation program grounded in shared reading. The intervention was delivered by a trained speech–language therapist within the inpatient pediatric hemato-oncology setting, with active caregiver involvement. Each session lasted approximately 30 min. The children received one to two sessions per week over a six-month period, with the frequency adjusted according to their clinical status, hospitalization conditions, and treatment schedules. The intervention employed age-appropriate interactive multisensory books selected according to developmental level and linguistic complexity. The materials included tactile, auditory, visual, and olfactory elements to facilitate engagement and multisensory stimulation [23]. Standardized materials were used across participants, with limited individual adaptations based on age and responsiveness. The sessions followed a structured shared reading protocol designed to promote joint attention, communicative engagement, and expressive language development. Strategies to foster interaction included optimal positioning and joint attention facilitation, while linguistic input was modulated through slowed speech rate, prosodic emphasis, repetition, and redundancy. To support expressive language production, evidence-based behavioral modeling techniques were applied, including prompting, fading, shaping, and reinforcement. Caregivers actively participated in all sessions and received structured counseling focused on responsive, child-centered communicative interaction styles [33]. Excessively directive or non-contingent interaction patterns were discouraged, whereas caregivers were guided toward tutorial interaction strategies aimed at supporting communication and language development [25]. Intervention fidelity was monitored through standardized checklists completed by the therapist and periodic clinical supervision to ensure consistency of protocol implementation among participants.

### 2.5. Statistical Analysis

All statistical analyses were performed using a significance level of *p* < 0.05. Descriptive statistics were computed for all demographic, clinical, and linguistic variables. Normality was assessed using the Shapiro–Wilk test, and non-parametric tests were applied due to significant deviations. Differences between language comprehension and production were examined using Wilcoxon signed-rank tests. Differences related to gender and diagnosis (solid tumors vs. hematologic malignancies) were analyzed using Mann–Whitney U tests. Effect sizes were calculated using the biserial rank correlation coefficient (r) to estimate the magnitude of observed effects. Associations between linguistic and cognitive measures were evaluated using Spearman’s rank correlation coefficients (ρ). To assess the robustness of significant associations, leave-one-out sensitivity analyses were conducted for all statistically significant correlations. Given the exploratory and pilot nature of the study, no correction for multiple comparisons was applied. Analyses were performed on the available cases for each measure, with sample sizes varying due to missing data across assessments.

## 3. Results

The study included 29 children under 36 months of age (Table 1). Of these, 16 were male (55%) and 13 were female (45%). The mean age at diagnosis was 20.76 months (SD = 9.52, range 2–36 months) and the mean time between diagnosis and assessment was 3.90 months (SD = 4.23, range 0–14 months). Seven children (24.1%) were also exposed to a second language. Participants were diagnosed with rhabdomyosarcoma, germinoma, low-grade glioma, or acute and myeloid lymphoblastic leukemia.

**Figure 1:**
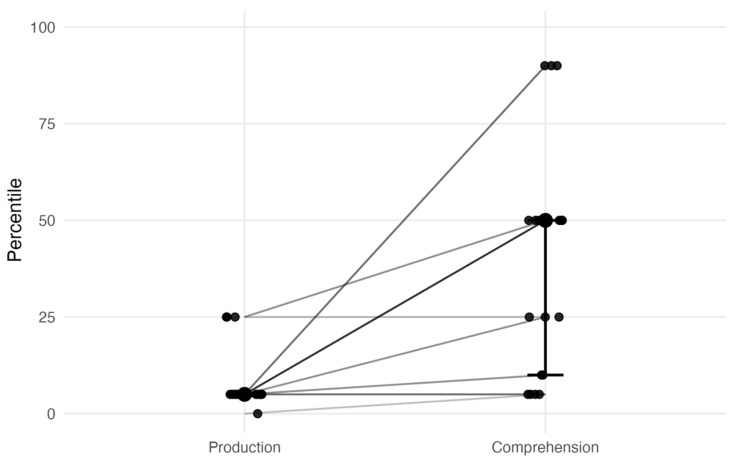
Paired comparison of baseline PinG lexical production and comprehension percentiles. Each line represents an individual participant and connects production and comprehension scores; black markers indicate the group median with interquartile range (IQR).

Mean PinG production scores were 39.25 (SD = 28.71), with a median of 50 and a range from 5 to 90. PinG comprehension was available for 20 children (69% of the sample), with a mean score of 39.25 (SD = 28.71, range 5–90, median = 50). PCGO scores were available for 19 children (66%), with a mean of 33.42 (SD = 24.55, range 5–90, median = 25). BAMF scores ranged from 1 to 6, with a mean of 3.55 (SD = 1.76, median = 4). GALS scores ranged from 0 to 4, with a mean of 1.72 (SD = 1.44, median = 1). Griffiths Communication & Language scores were available for 21 children (72%), with a mean of 40.76 (SD = 32.52, range 2–93, median = 21). For lexical skills assessed by the PinG, participants showed significantly higher performance in comprehension compared with production. This difference was confirmed by a Wilcoxon signed-rank test (W = 136, *p* < 0.001, r = 0.84; *N* = 20; Figure 1).

Correlation analyses (Spearman’s ρ) showed that lexical production (PinG) was positively associated with articulation (BAMF; ρ = 0.557, *p* = 0.0108; *N* = 20), and this association remained statistically significant in a leave-one-out sensitivity analysis (worst-case: ρ = 0.483, *p* = 0.036; *N* = 19 per iteration), whereas the association between morphosyntactic production (GALS) and morphosyntactic comprehension (PCGO) was moderate (ρ = 0.472, *p* = 0.0411; *N* = 19) but was not consistently preserved across leave-one-out iterations (worst-case: ρ = 0.393, *p* = 0.106; *N* = 18), and the correlation between GALS and overall communication abilities (Griffiths) did not reach statistical significance (ρ = 0.377, *p* = 0.0921; *N* = 21) and remained non-significant in the leave-one-out analyses (worst-case: ρ = 0.293, *p* = 0.210; *N* = 20). No other correlations reached statistical significance, suggesting that these associations may be domain-specific and potentially reflect a greater vulnerability of expressive language skills among young children with hemato-oncological conditions.

As shown in Table 2, a significant difference emerged for PinG lexical comprehension, with males scoring lower than females, while no statistically significant gender differences were observed for the other measures (all *p* > 0.05), with trend-level effects for PinG lexical production and Griffiths communication.

**Figure 2:**
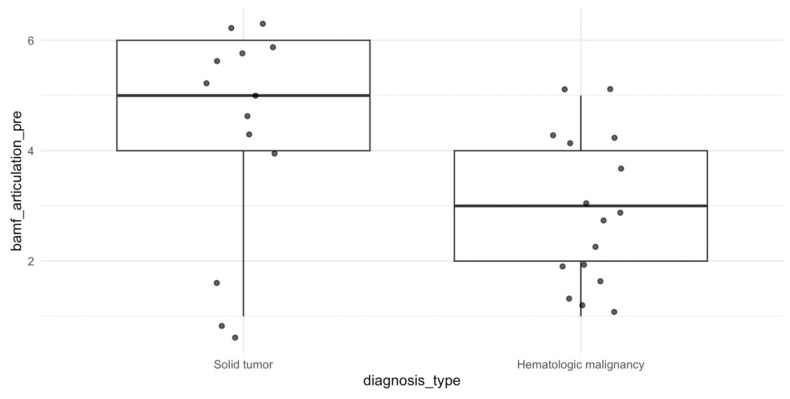
Distribution of BAMF—articulation scores by diagnosis type (solid tumor vs. hematologic malignancy).

Independent-samples comparisons by pathology type (solid tumors vs. hematologic malignancies) were conducted (Table 3). Children with solid tumors showed higher articulation scores (BAMF) than those with hematologic malignancies, as shown in Figure 2. A trend toward a difference was observed regarding morphosyntactic production (GALS), with higher scores in the solid tumor group. No significant differences were found for PinG comprehension or production, PCGO, or overall communication abilities (Griffiths) (all *p* > 0.05).

**Figure 3:**
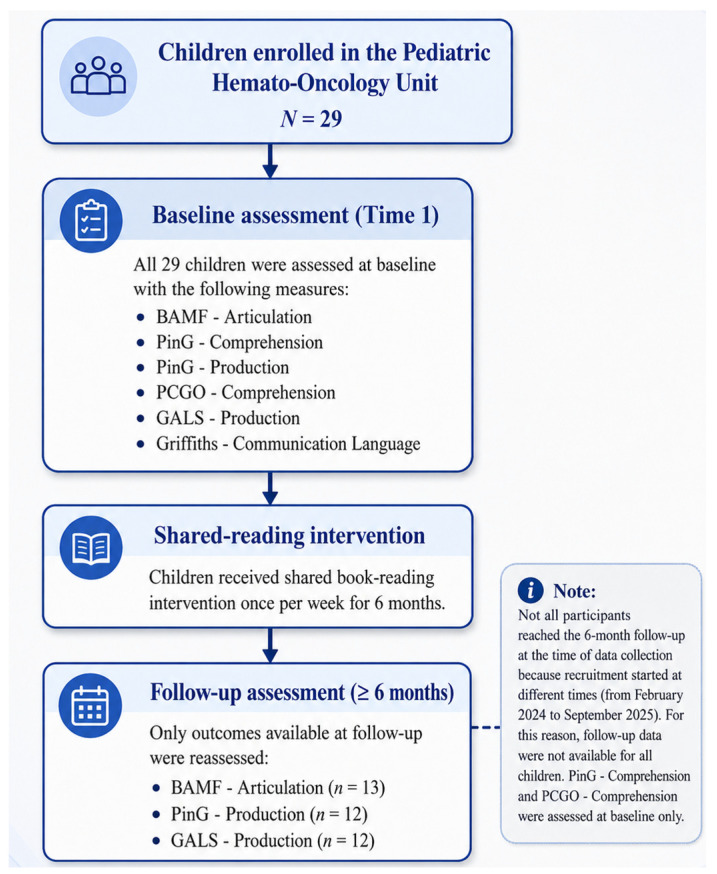
Flowchart depicting participant progression throughout the study.

Of the initial sample of 29 children, all those attending the day hospital or receiving inpatient care in the Pediatric Hemato-Oncology Unit received communicative–linguistic stimulation through shared book reading. However, only children who had completed six months of intervention were eligible for the follow-up evaluation. Because participants were recruited at different time points during the study period, not all children were able to complete the six-month intervention phase within the study timeframe. Therefore, the data reported here refer to the subgroup of children (*N* = 13) who completed the six-month treatment period and were consequently re-evaluated, as shown in Figure 3. However, paired sample sizes varied across outcome measures due to occasional missing follow-up data.

To evaluate change over time, Wilcoxon signed-rank tests were used, limited to measures available in both assessments; PinG comprehension and PCGO were assessed only at baseline and were therefore not included, while Griffiths scores could not be analyzed due to an insufficient number of paired observations (*N* = 2). As shown in Table 4, articulation improved significantly, as did morphosyntactic production (GALS) and lexical production (PinG production). Statistical analyses confirmed that these gains were significant, suggesting that the early communicative–linguistic intervention may have had a positive effect on expressive skills.

## 4. Discussion

The findings of the present study appear to be consistent with existing literature pointing toward the central role of an interactive, verbally rich environment in supporting early language development [34]. In particular, the results suggest that the quality of linguistic input offered to the child—especially during sensitive developmental periods—plays a more pivotal role than the specific materials used or the frequency of activities [2,11,12]. Activities that promote children’s engagement and enjoyment appear to elicit richer and more complex caregiver input, thereby increasing learning opportunities [13,14]. This aspect is particularly relevant for children with hemato-oncological conditions, who often experience reduced opportunities for spontaneous interaction due to prolonged hospitalization, intensive medical treatments, and limited peer contact [35].

The children included in this study were exposed to multiple cumulative risk factors for language and communication delay, including extended hospital stays, frequent medical procedures, and increased exposure to passive digital media [6,7]. These environmental constraints could potentially explain the vulnerability observed in expressive language domains. Overall, the data tend to support the hypothesis that hospital-related deprivation may not affect all linguistic components equally but rather appear to have a disproportionate impact on expressive skills, which arguably require more active practice and interaction.

Consistent with previous research [8,10], the data suggest a trend toward dissociation between language comprehension and production in this cohort. With regard to lexical abilities, children demonstrated significantly higher performance in comprehension than in production. These findings may suggest a specific vulnerability of expressive language, rather than a generalized linguistic impairment, and appear to be consistent with commonly reported developmental patterns in children under 36 months, particularly in populations exposed to adverse environmental conditions [33,34]. Articulatory skills also appear to be below expectations relative to chronological age. The mean BAMF score reflects an immature articulatory repertoire, which could potentially limit the development of expressive language. Correlation analyses showed a significant positive association between articulatory skills and lexical production, suggesting that children with better articulatory control tended to demonstrate more advanced expressive vocabulary. This observation is in line with theoretical models proposing an interdependence between motor speech development and lexical growth [36,37].

A moderate association has been observed between morphosyntactic comprehension and production, potentially reflecting the close interconnection between comprehension and production of syntactic structures during the early stages of development, even in contexts where overall expressive production is limited [38]. Taken together, these correlations may support the idea that language development functions as a highly integrated system, in which lexical, morphosyntactic, articulatory, and cognitive–communicative components can influence one another [38]. In line with previous research, lexical and morphosyntactic skills may represent fundamental elements for overall language development and could therefore be considered priority targets for early intervention in children under 36 months of age [39].

With regard to demographic variables, males performed worse than females on lexical comprehension, whereas no statistically significant gender differences were observed in morphosyntactic production and articulation. Although trend-level effects emerged for lexical production and communication according to Griffiths, these did not reach statistical significance. Previous research has reported a small but relatively consistent female advantage in early lexical production, with girls demonstrating a larger expressive vocabulary and faster vocabulary growth than boys across all languages and cultural contexts [40]. Considering these findings, the present results may be interpreted within the framework of the PinG assessment, which is normed for both age and sex. The use of sex-specific normative data reflects well-established developmental patterns indicating that males, on average, may exhibit a smaller expressive vocabulary compared to females during the first three years of life [28,36]. These differences tend to emerge during the second year of life and are hypothesized to reflect a combination of biological predispositions and interactional dynamics, rather than differences in linguistic input alone [40].

Regarding pathology type, previous studies have reported highly variable prevalence rates of communication difficulties in children with pediatric brain tumors or leukemia, ranging from 14% to 100% depending on the tumor side, treatment modality, and the timing of the assessment [9]. In the present study, children with solid tumors showed significantly better articulatory skills than those with liquid tumors, with a marginally significant advantage also observed in morphosyntactic production. This pattern may partly reflect differences in clinical management between liquid and solid tumors. In our sample, children with hematologic cancers appeared to have a higher overall risk of complications and mortality compared to those with solid tumors, potentially requiring longer and more restrictive hospitalizations [41]. Such conditions may reduce opportunities for social interaction and spontaneous language practice. Since articulatory development is believed to depend largely on frequent motor practice and interactive feedback, these constraints could disproportionately affect speech production, while receptive language skills may remain relatively preserved [36,37].

Finally, the longitudinal data would support the hypothesis that early communicative–linguistic intervention delivered by an SLT may be associated with improved language outcomes in infants and toddlers with hemato-oncological conditions. In the subgroup of children re-evaluated after six months of intervention, improvements were observed in articulation, morphosyntactic and lexical production. These findings would suggest that structured, interactive stimulation, such as shared book reading, may mitigate the negative impact of hospitalization on expressive language development [13], acting as a protective and compensatory factor by promoting enriched caregiver–child interaction, increased language exposure, and more frequent episodes of shared attention [23]. These results would underscore the potential role of the speech–language therapist within pediatric hemato-oncology units. In line with “The Clinical Practice Guideline for the Management of Communication and Swallowing following Childhood Brain Tumour and Leukaemia” [20], systematic assessment and ongoing monitoring of language functions should be implemented in children with hemato-oncological diagnoses. Evidence of possible delayed declines in receptive and higher-order language abilities following an initial period of post-treatment stability would further support the need for longitudinal monitoring of linguistic performance in children exposed to chemotherapy and related medical treatment [8]. Early and targeted SLT involvement, encompassing both evaluation and intervention, may be crucial for the timely identification of emerging communicative vulnerabilities, support of language development, and promotion of quality of life and communicative participation throughout and beyond medical treatment [9,13].

### 4.1. Limits

The present study is subject to several limitations that should be considered when interpreting the findings. First, the sample size was relatively small, as participant recruitment was limited to children enrolled in the “Stai Bene 3.0 Plus” and “Stai Bene 4.0” projects. At present, SLT services are not integrated as a permanent component of the Pediatric Hemato-Oncology Unit; instead, access to SLT assessment and intervention is restricted to patients who voluntarily participate in these externally funded projects.

A key limitation of the study is the variability in the intensity and consistency of the SLT intervention, which was influenced by patients’ clinical conditions and competing medical priorities. The absence of a control group further limits the ability to draw definitive conclusions about the causal effects of the intervention, as observed improvements may partly reflect spontaneous language development rather than gains specifically attributable to the intervention. Moreover, complete data collection was not feasible for all participants due to medical instability and follow-up assessments performed at centers closer to the child’s home, thereby limiting the availability of comprehensive evaluation data.

### 4.2. Future Perspectives

Future research should extend follow-up assessments beyond the immediate post-treatment period to determine whether the observed language gains are maintained over time rather than merely reflecting short-term effects. Systematic reassessment at least six months post-intervention would provide stronger evidence regarding the persistence of language improvements and the potential impact of ongoing environmental or treatment-related factors. The present study focuses on a highly specific and clinically vulnerable population—children under 36 months undergoing hemato-oncological treatment—who remain largely underrepresented in the literature. Although the sample size is modest (*N* = 29), it is noteworthy given the rarity and clinical complexity of this patient group. Building on these findings, future studies employing larger samples, well-matched control groups, and longitudinal designs are warranted to differentiate intervention-specific effects from natural developmental trajectories and to further elucidate the long-term efficacy of communicative interventions in pediatric hemato-oncology populations.

## 5. Conclusions

The findings of this study are consistent with existing literature reporting the presence of language difficulties in pediatric hemato-oncology populations. Early interventions, such as shared book reading, may support language development in children under 36 months and help prevent the consolidation of delays. Integrating SLT into pediatric oncology teams is crucial for the timely identification of communicative difficulties and the implementation of early intervention, thereby promoting language development, social reintegration, and overall quality of life.

## Figures and Tables

**Table 1:** Sample characteristics of the study population (*N* = 29). Values are presented as mean (SD) or *n* (%).

Sample Characteristics	Total 29 (%)
Age at diagnosis, mean (SD)	20.76 (9.52)
Age at assessment, mean (SD)	24.8 (8.77)
Gender	
Male	16 (55%)
Female	13 (45%)
Second language exposure	
Yes	7 (24.1%)
No	22 (75.9%)
Pathology type	
Hematologic	16 (55%)
Solid	13 (45%)

**Table 2:** Comparison of language and communication measures between female and male participants at baseline. Values are reported as median [IQR] with group-specific sample sizes. Effect sizes are reported as r. *N* varies across measures due to missing data.

	Male	Female	*N*	U	*p*	r
BAMF—Articulation	4 [2, 5] (*n* = 16)	4 [2, 4] (*n* = 13)	29	93	0.62	0.09
PinG—Comprehension	50 [37.5, 50] (*n* = 11)	10 [5, 50] (*n* = 9)	20	24.5	0.05	0.44
PinG—Production	5 [5, 15] (*n* = 11)	5 [5, 5] (*n* = 9)	20	32	0.06	0.43
PCGO—Comprehension	25 [25, 50] (*n* = 11)	25 [8.75, 31.25] (*n* = 8)	19	31.5	0.27	0.25
GALS—Production	1.5 [0.75, 3] (*n* = 16)	1 [1, 2] (*n* = 13)	29	97.5	0.77	0.05
Griffiths—Communication Language	61 [18.5, 78] (*n* = 11)	15 [9, 40.5] (*n* = 10)	21	28	0.06	0.42

**Table 3:** Comparison of baseline language and communication measures by diagnosis type (solid tumors vs. hematologic malignancies). Values are reported as median [IQR] with group-specific sample sizes. Effect sizes are reported as r. *N* varies across measures due to missing data.

	Solid	Hematologic	*N*	U	*p*	r
BAMF—Articulation	5 [4, 6] (*n* = 13)	3 [2, 4] (*n* = 16)	29	157	0.02	0.44
PinG—Comprehension	25 [15, 50] (*n* = 7)	50 [10, 50] (*n* = 13)	20	39	0.59	0.12
PinG—Production	5 [5, 15] (*n* = 7)	5 [5, 5] (*n* = 13)	20	57.5	0.17	0.31
PCGO—Comprehension	25 [17.5, 70] (*n* = 7)	25 [25, 31.25] (*n* = 12)	19	49.5	0.50	0.15
GALS—Production	2 [1, 4] (*n* = 13)	1 [0, 2] (*n* = 16)	29	145	0.07	0.34
Griffiths—Communication Language	61 [16, 86] (*n* = 9)	18.5 [11.75, 52.25] (*n* = 12)	21	72.5	0.19	0.29

**Table 4:** Pre–post comparisons for the shared-reading intervention subgroup (participants reassessed at follow-up). Values are reported as median [IQR] at each time point with paired sample size (*N*). Effect sizes are reported as r.

	*N*	Pre	Post	W	*p*	r
BAMF—Articulation	13	3 [2, 4] (*n* = 13)	5 [3, 6] (*n* = 13)	55	0.0026	0.84
PinG—Production	12	5 [5, 5] (*n* = 12)	10 [5, 28.12] (*n* = 12)	36	0.0061	0.79
GALS—Production	12	1 [1, 1.5] (*n* = 12)	2 [2, 4.25] (*n* = 12)	55	0.0027	0.87
Griffiths—Communication Language	2	54 [36, 72] (*n* = 2)	14 [8.5, 19.5] (*n* = 2)	NA	NA	NA

Note. Effect size r: ~0.10 small, ~0.30 moderate, ~0.50 large.

## Data Availability

The de-identified dataset, codebook, and README are available in an OSF repository (view-only link): https://osf.io/pe765/overview?view_only=4e633afff10f4c468c540358ff4cac94 (accessed on 30 March 2026).

## Abbreviations

The following abbreviations are used in this manuscript:

SLT	Speech-language therapist
CNS	Central nervous system
PinG	Parole in Gioco
PCGO	Prova di Comprensione Grammaticale con Oggetti
GALS	Griglia di analisi del linguaggio spontaneo
BAMF	Brief Assessment of Motor Function
